# The safety and efficacy of endoscopic endonasal approach in the treatment of recurrent craniopharyngioma: A protocol for systematic review and meta-analysis

**DOI:** 10.1097/MD.0000000000022995

**Published:** 2020-12-04

**Authors:** Pengtao Li, Aximujiang Axier, Shaoshan Li, Kai Zhou, Jingwei Yun, Huayi Wang, Tingrong Zhang

**Affiliations:** Department of Neurosurgery, The First Affiliated Hospital of Xinjiang Medical University, Urumqi, Xinjiang Uygur Autonomous Region, China.

**Keywords:** efficacy, endoscopic endonasal approach, meta-analysis, protocol, recurrent craniopharyngioma, safety, systematic review

## Abstract

**Background:**

Craniopharyngioma is the most challenging brain tumor with a high recurrence rate. Some scholars have shown that endoscopic endonasal approach (EEA) can achieve a higher total tumor resection rate and significantly reduce the incidence of complications and mortality. However, there is still no consensus on the surgical approach for recurrent craniopharyngioma. The purpose of this study is to evaluate the safety and efficacy of EEA in the treatment of recurrent craniopharyngioma.

**Methods:**

We will search 7 electronic databases (PubMed, EMBASE, Web of Science, the Cochrane Library, PsycINFO, AMED, Scopus) to collect related randomized controlled trials (RCTs). The resection rate, recurrence rate and progression-free survival rate will be regarded as the primary outcome, and the incidence of complications will be regarded as the secondary outcome. Endnote Software X9.0 will be used to filter articles, Review Manager Software 5.2 and STATA software 16.0 will be used for analysis and synthesis.

**Results:**

We will integrate existing studies to assess the safety and efficacy of EEA in the treatment of recurrent craniopharyngioma.

**Conclusion:**

Our study will provide EEA as an effective and safe treatment for recurrent craniopharyngioma.

**Registration number:**

International Prospective Register of Systematic Reviews (PROSPERO): CRD42020199860

## 1 Introduction

Craniopharyngioma is a benign brain tumor originating from the residual epithelial cells of the embryonic Rathke cyst.^[1]^ Although it is a benign tumor, due to the calcification in the capsule and close to the hypothalamus, pituitary gland, optic nerve and carotid artery, and other important nerve vessels, it is difficult to complete surgical resection. It is called as the most challenging brain tumor.^[2–4]^ According to literature reports, the recurrence rate after total resection is 0% to 26%, and the recurrence rate after subtotal resection is as high as 75% to 100%.^[5–7]^ When the tumor recurs, it can be controlled by reoperation or radiotherapy, but radiotherapy is less effective in controlling the growth of cystic tumors such as craniopharyngioma.^[8–10]^ Therefore, for the treatment of recurrent craniopharyngioma, reoperation as the primary adjuvant radiotherapy is recommended to achieve the goal of total resection or control of tumor growth.^[11,12]^

In recent years, endoscopic endonasal approach (EEA) has been widely used in the treatment of craniopharyngioma. Even if the tumor invades the supra sella cisterna and the third ventricle upward, EEA can also replace the traditional craniotomy approach to achieve good tumor resection.^[13]^ Some scholars have shown that EEA can achieve a higher total tumor resection rate and greatly reduce the incidence of complications and mortality.^[14–17]^ However, there is still no consensus on the surgical approach for recurrent craniopharyngioma. The purpose of our study is to assess the safety and efficacy of EEA in the treatment of recurrent craniopharyngioma.

## 2 Methods and analysis

### 2.1 Study registering

The protocol registration number of the study is CRD42020199860 (PROSPERO). We will strictly follow the Preferred Reporting Items for Systematic Review and Meta-Analysis Protocols (PRISMA-P) guidelines.^[18]^

### 2.2 Eligibility criteria

We will develop inclusion and exclusion criteria based on the five main principles of PICOS.

#### 2.2.1 Type of participants

The participants in the study were pathologically confirmed as craniopharyngioma at the initial operation, and were diagnosed as recurrent craniopharyngioma by cranial imaging examination before the reoperation. The diagnosis of recurrent craniopharyngioma was based on Liubinas et al.^[19]^ All participants must be older than 18 years old at first diagnosis.

#### 2.2.2 Type of interventions and comparators

Our intervention is EEA in the treatment of recurrent craniopharyngioma. And our comparator is craniotomy for recurrent craniopharyngioma.

#### 2.2.3 Types of outcome measurements

##### 2.2.3.1 Primary outcome

1. The total resection rate (number of participants with total resection/ total number of participants in this group);
2. Progression-free survival (PFS) (the time between treatment and tumor progression or death from any cause);
3. The recurrence rate (number of people who relapsed during follow-up/ total number of participants in this group).

##### 2.2.3.2 Secondary outcomes

1. Endocrine disorders rate (number of patients with endocrine disorders/total number of patients in this group);
2. Visual impairment rate (number of patients with visual impairment/total number of patients in this group);
3. Mortality rate (number of deaths/total number of patients in this group);

#### 2.2.4 Exclusion criteria

1. Participants with the unclear diagnosis;
2. The first diagnosis was less than 18 years old;
3. Endoscopy was not used as the primary treatment;
4. Insufficient public data to estimate the result rate;
5. The studies where full text was unavailable.

### 2.3 Search methods for identification of studies

#### 2.3.1 Electronic data sources

The following seven electronic databases will be searched from inception to 25 August 2020: PubMed, EMBASE, Web of Science, the Cochrane Library, PsycINFO, AMED, Scopus.

#### 2.3.2 Other resources

For a more comprehensive review and collection of articles. We will also search for ongoing trials (the World Health Organization's international clinical trial registration platform). In addition, relevant grey literature (Health Management Information Database, OpenSIGLE Database, and the National Technical Information Service) will also be reviewed and collected.

### 2.4 Search strategy

We will combine keywords and text words as search strategies. The search terms will be expanded around: endoscopic endonasal approach, recurrent craniopharyngioma, and RCTs. There are no restrictions on publication date and language. As shown in Table 1, taking PubMed as an example of the search strategy, in addition, the search strategy will be changed according to the characteristics of different databases.

**Table 1:** Search strategy used in PubMed database.

Number	Search items
1	endoscopic endonasal [MeSH Terms]
2	endoscopic endonasal [Title/Abstract] OR endoscopic transsphenoidal [Title/Abstract] OR transsphenoidal [Title/Abstract]
3	1 OR 2
4	craniopharyngioma [MeSH Terms]
5	craniopharyngioma [Title/Abstract] OR craniopharingiomas [Title/Abstract] OR craniopharingioma^∗^ [Title/Abstract] OR Neoplasm, Rathke's Cleft [Title/Abstract] OR Neoplasm, Rathkes Cleft [Title/Abstract] OR Rathke's Pouch Tumor [Title/Abstract] OR Rathkes Pouch Tumor [Title/Abstract] OR Tumor, Rathke's Pouch [Title/Abstract] OR Rathke Pouch Tumor [Title/Abstract] OR Tumor, Rathke Pouch [Title/Abstract] OR Rathke's Cleft Neoplasm [Title/Abstract] OR Rathkes Cleft Neoplasm [Title/Abstract] OR Neoplasm, Rathke Cleft [Title/Abstract] OR Rathke Cleft Neoplasm [Title/Abstract] OR Craniopharyngioma, Papillary [Title/Abstract] OR Craniopharyngiomas, Papillary [Title/Abstract] OR Papillary Craniopharyngioma [Title/Abstract] OR Papillary Craniopharyngiomas [Title/Abstract] OR Craniopharyngioma, Child [Title/Abstract] OR Child Craniopharyngioma [Title/Abstract] OR Child Craniopharyngiomas [Title/Abstract] OR Craniopharyngiomas, Child [Title/Abstract] OR Craniopharyngioma, Adamantinous [Title/Abstract] OR Adamantinous Craniopharyngioma [Title/Abstract] OR Adamantinous Craniopharyngiomas [Title/Abstract] OR Craniopharyngiomas, Adamantinous [Title/Abstract] OR hypophyseal duct tumor [Title/Abstract] OR hypophyseal duct tumors [Title/Abstract] OR adamantinoma [Title/Abstract] OR adamantinomas [Title/Abstract] OR Craniopharyngeal duct tumour [Title/Abstract] OR Adamantinomatous tumour [Title/Abstract]
6	4 OR 5
7	recurrence [MeSH Terms]
8	ecurrence [Title/Abstract] OR tumor recurrence [Title/Abstract] OR neoplasm recurrence [Title/Abstract] OR local recurrence [Title/Abstract] OR invasiveness OR metastasis OR cocarcinogenesis
9	7 OR 8
10	randomized controlled trial [Publication Type]
11	randomized [Title/Abstract]
12	randomly [Title/Abstract]
13	10 OR 11 OR 12
14	3 AND 6 AND 9 AND 13

### 2.5 Data collection

#### 2.5.1 Selection of studies

**Figure 1:**
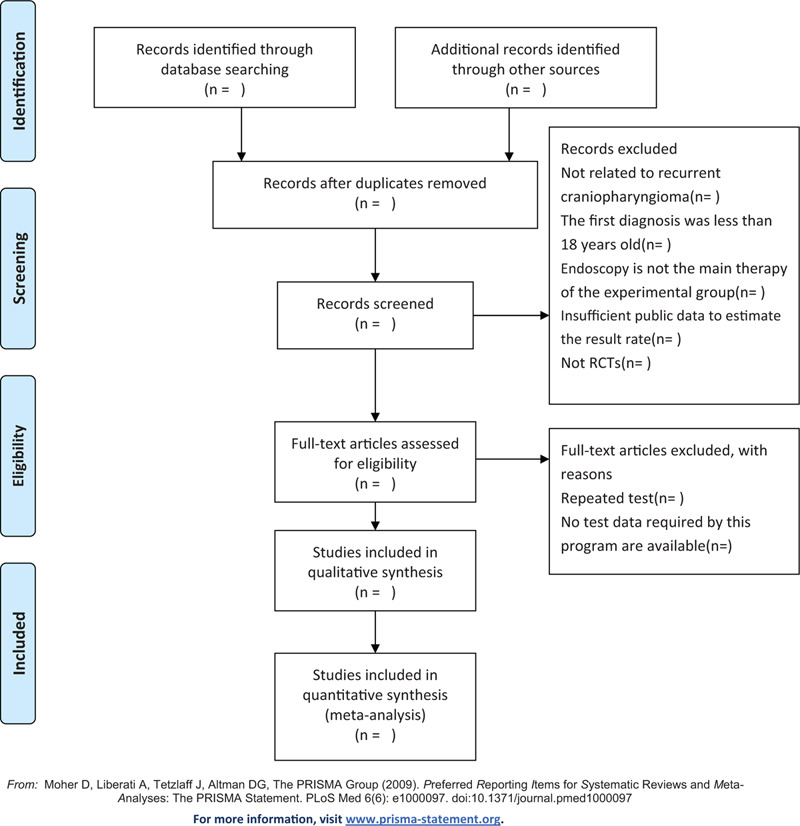
The Preferred Reporting Items for Systematic Reviews and Meta-Analyses Protocols flow diagram of the study selection process. *From:* Moher D, Liberati A, Tetzlaff J, Altman DG, The PRISMA Group (2009). *P*referred *R*eporting *I*tems for *S*ystematic Reviews and *M*eta-*A*nalyses: The PRISMA Statement. PLoS Med 6(6): e1000097. doi:10.1371/journal.pmed1000097. For more information, visit www.prisma-statement.org.

We will import the extracted study into the Endnote Software X9.0 to remove duplicates. According to our inclusion and exclusion criteria, the title and abstract of the study were selected independently by two researchers (PTL and AA). After that, the full text will be served as a second filter. Two researchers (PTL and AA) will cross-check the included studies, and if there is any disagreement, a third researcher (SSL) will participate. Our PRISMA-P flow diagram (Fig. 1) will show the detailed screening process.

#### 2.5.2 Data extraction and management

Two other researchers (KZ and JWY) will sift through all the articles retrieved in the above database to find those that meet the requirements. If there is any disagreement, read the full text and the third researcher (HYW) will participate. The data will be extracted independently by two researchers (KZ and JWY) using pre-designed data extraction forms (basic information, inclusion exclusion criteria, participant information, trial and control group details, results, conclusions, follow-up, adverse events). A third researcher will verify the data. Contact the author of the study for more information if necessary.

#### 2.5.3 Assessment of risk of bias in included studies

Methodological quality of eligible studies will be evaluated independently by 2 researchers (PTL and SSL) according to the Cochrane Intervention Systematic Review Manual. The following characteristics will be assessed: selection, performance, attrition, detection, reporting, and other sources of bias.

According to the evaluation of the study, the bias was classified as “low risk”, “high risk” or “unclear risk”. When a disagreement arises, it is resolved through discussion, or a third researchers (AA) may be involved.

### 2.6 Data synthesis

Meta-analysis was performed using Review Manager Software 5.2 and STATA software 16.0. Odds ratio (OR) and average difference (MD) were used as 95% confidence intervals for the dichotomous data and the continuous data. Statistical heterogeneity between studies was tested by calculating Higgins *I*^2^ values or using the *x*^2^ test. If there is significant statistical heterogeneity in the test, a random effect model will be used for synthesis. Otherwise, the data will be processed with a fixed effect model. If significant statistical heterogeneity exists, descriptive analysis will be performed.

#### 2.6.1 Measures of treatment effect

We will use the mean difference assessment Progression-free survival (PFS), to analyze the ratio to assess total resection, recurrence, endocrine disorders, visual impairment, and mortality. All take 95% confidence intervals (CIs).

#### 2.6.2 Management of missing data

If the data is insufficient or missing, the corresponding author of the study will be contacted. If accurate data are still not available after contacting the authors, and these studies will be excluded.

#### 2.6.3 Assessment of heterogeneity

We made qualitative analysis by comparing the characteristics of the included studies, and used *I*^2^ test and *X*^2^ test for quantitative analysis of heterogeneity. *I*^2^ > 25%, *I*^2^ > 50%, and *I*^2^ > 75% were defined as moderate, substantial, and considerable heterogeneity, respectively. If *I*^2^ > 50%, it will be considered as significant homogeneity.

#### 2.6.4 Assessment of reporting biases

We assessed publication bias by selecting either funnel plots or the Egger test. Funnel plots will be selected when 10 or more RCTs meet the requirements for inclusion. Otherwise, we will use STATA software 16.0 to perform the Egger test.

#### 2.6.5 Subgroup analysis

To investigate potential heterogeneity across studies, we will conduct subgroup analysis according to different time points, tumor size, pathological type, first postoperative radiotherapy and chemotherapy, and observe the results.

#### 2.6.6 Sensitivity analysis

For studies with a risk of bias, data, and sample size deficiencies, sensitivity analyses are performed to assess robustness if statistically significant heterogeneity exists.

### 2.7 Grading the quality of evidence

The quality of evidence was evaluated from five aspects of limitation of study design, inconsistency, indirectness, imprecision, and bias of publication. According to the Grading of Recommendations Assessment, Development, and Evaluation, the quality grade was divided into four grades: very low, low, moderate, and high.

### 2.8 Ethics and dissemination

Considering that our study is not related to individual patient data, ethical approval is not necessary. Our results will be presented in a peer-reviewed journal or related conference to evaluate the safety and efficacy of endoscopic endonasal approach in the treatment of recurrent craniopharyngioma.

## 3 Discussion

The annual incidence of craniopharyngioma is 1.3 per million people.^[2]^ Although it is a benign tumor, the postoperative recurrence rate, mortality rate and complication rate are high, seriously affecting the life and quality of life of the patients.^[20–23]^ In recent years, with the continuous application and development of EEA in craniopharyngioma patients, it has gradually replaced traditional craniotomy and is highly praised for its unique advantages such as less trauma and fewer postoperative complications.^[24–27]^ Recurrent craniopharyngioma further increases the difficulty of surgery due to scar and adhesion after the previous operation, and the optimal surgical method is still controversial. EEA may be an effective and safe treatment method. But there is no clear conclusion. Strictly speaking, a systematic review and meta-analysis of existing RCTs were conducted to assess the safety and efficacy of EEA, with the aim of providing evidence for clinical practice and facilitating future research, according to the Cochrane Manuals systematic review of interventions.

## Author contributions

**Conceptualization:** Pengtao Li, Aximujiang Axier, Shaoshan Li.

**Data curation:** Kai Zhou, Huayi Wang.

**Funding acquisition:** Shaoshan Li.

**Methodology:** Pengtao Li.

**Software:** Jingwei Yun.

**Supervision:** tingrong zhang.

**Writing – original draft:** Pengtao Li, Aximujiang Axier, Shaoshan Li.
